# A 27-Month-Old Boy with Polyuria and Polydipsia

**DOI:** 10.1155/2018/4281217

**Published:** 2018-08-23

**Authors:** Yvonne Lee, Erica Winnicki, Lavjay Butani, Stephanie Nguyen

**Affiliations:** ^1^Department of Pediatrics, Section of Endocrinology, Kaiser Permanente Oakland Medical Center, Oakland, CA, USA; ^2^Department of Pediatrics, Section of Nephrology, University of California, San Francisco, San Francisco, CA, USA; ^3^Department of Pediatrics, Section of Nephrology, University of California, Davis, Sacramento, CA, USA

## Abstract

Psychogenic polydipsia is a well-described phenomenon in those with a diagnosed psychiatric disorder such as schizophrenia and anxiety disorders. Primary polydipsia is differentiated from psychogenic polydipsia by the lack of a clear psychotic disturbance. We present a case of a 27-month-old boy who presented with polyuria and polydipsia. Laboratory studies, imaging, and an observed water deprivation test were consistent with primary polydipsia. Polydipsia resolved after family limited his fluid intake and began replacing water drinking with other transition objects and behaviors for self-soothing. This case highlights the importance of water deprivation testing to differentiate between causes of polyuria, thereby avoiding misdiagnosis and iatrogenic hyponatremia. Secondly, primary polydipsia can result during the normal stages of child development without overt psychiatric disturbances.

## 1. Case Description

A 27-month-old boy was referred for a second opinion regarding polyuria and polydipsia of sudden onset four months prior to presentation. He drank between 3 and 4 L of water per day and had frequent heavy wet diapers, decreased appetite, and a 1-pound weight loss. He had no prior episodes of dehydration or any preceding or intercurrent illnesses. He had no prior medical history, met normal developmental milestones, and was not taking any prescribed or over-the-counter medications. There was no family history of diabetes insipidus. Social history was remarkable for the mother returning to work prior to the onset of symptoms.

Prior evaluation was significant for normal serum sodium, glucose, blood urea nitrogen (BUN), creatinine, adrenocorticotropic hormone (ACTH), thyroid-stimulating hormone (TSH), thyroxine level, insulin-like growth factor (IGF), cortisol, erythrocyte sedimentation rate (ESR), and prolactin. The random arginine vasopressin (AVP) level was 1.4 pg/mL (normal range 1–13.3 pg/mL) with a random urine osmolality of 285 mOsm/kg. A head MRI did not reveal any pituitary or other intracranial pathologies. A renal ultrasound showed a right kidney with a duplicated collecting system with mild prominence of the lower pole of the renal pelvis. An informal water deprivation test was conducted at home, and parents were instructed to limit water intake at home overnight and to return for laboratory evaluation in the morning. Urine osmolality was 683 mOsm/kg after 12 hours of water deprivation. However, mother stated that, at the end of the water deprivation, she had to give him water to stimulate diuresis. No serum osmolality or sodium levels were collected. He was diagnosed with partial DI and started on oral desmopressin (DDAVP). The dose of DDAVP was titrated up to 0.2 mg twice daily for effect, his polyuria and polydipsia resolved, his appetite improved, and he began to gain weight.

Vital signs were normal, with weight-for-age at the 45^th^ percentile, height-for-age at the 57th percentile, and body surface area 0.57. Physical exam was remarkable only for a tired-appearing toddler. Chemistries performed were remarkable for serum sodium of 121 mEq/L. DDAVP was discontinued, and the serum sodium normalized to 138 mEq/L 24 hours later.

He was admitted to the hospital for a water deprivation test. After eight hours of observed water deprivation, urine osmolality increased from 270 mOsm/kg to 753 mOsm/kg. During this observed period, urine output was 4 ml/kg/hour and total weight loss was 160 grams. Serum sodium was 139 mEq/L and 140 mEq/L and serum osmolality was 298 mOsm/kg and 294 mOsm/kg, before and after water deprivation, respectively. He was discharged home with fluid restriction to 1.5 L/day. Since discharge from the hospital, parents were able to reduce his water intake with resolution of polyuria. However, he continued to ask for water to help him fall asleep at night. Retrospectively, his mother suspects that water drinking was his method for self-comfort following her return to work.

## 2. Diagnosis

He was diagnosed with primary polydipsia.

## 3. Discussion

Polydipsia and polyuria are defined as excessive fluid intake and >2000 ml/m^2^/day of urine output, respectively [1]. The differential diagnosis of polydipsia with polyuria includes primary polydipsia (PP), central diabetes insipidus (CDI), nephrogenic diabetes insipidus (NDI), and others (Table 1). Primary polydipsia can be further separated into thirst driven or non-thirst driven as seen in psychiatric illness (psychogenic polydipsia) or habitual drinkers. If initial history, physical exam, and laboratory assessment are concerning for either CDI, NDI, or PP, a water deprivation test should be performed to differentiate between the three possible diagnoses (Table 2) [1, 2].

After formal water deprivation testing, the patient demonstrated an increased urine osmolality with normal serum osmolality and normal serum sodium. The urine osmolality increased to >750 mOsm/kg without elevations in serum sodium or serum osmolality, which was diagnostic for PP (Table 2). DDAVP is normally given during the water deprivation test to differentiate between CDI and NDI, but since the patient demonstrated an increase in urine osmolality, DI was ruled out and DDAVP was not given.

In this case, the prior informal water deprivation testing at home was misleading and highlights the challenges of this approach. Concomitant plasma osmolality should be measured for the interpretation of urine osmolality to ensure that after water deprivation plasma osmolality is sufficient to stimulate vasopressin release. Surreptitious fluid intake can lead to misinterpreted results, thus resulting in hyponatremia after DDAVP and unrestricted water intake. PP in infants and children is an unusual condition that results in excessive fluid intake not caused by an intrinsic endocrine, renal, or metabolic disease [3]. PP is preferable over psychogenic polydipsia to describe compulsive water drinking unless there is a clear psychotic disturbance. Psychogenic polydipsia is commonly described in adults with psychiatric diagnoses [4] and occurs in 6%–20% of adult psychiatric patients, in particular, those with schizophrenia [5] and anxiety disorders [5]. It has also been reported following traumatic brain injury [6]. Primary polydipsia [3, 7] and psychogenic polydipsia [8, 9] are infrequently described in infants and children, and often, the distinction between the two is difficult to make [8]. PP may also be difficult to differentiate from DI, as some forms of DI present gradually and may initially seem more like PP or partial CDI [10]. In addition, PP often results in impaired urinary concentrating ability, which can complicate the workup. In animal models, this has been found to be due to decreased aquaporin-2 in the collecting duct and decreased aquaporin-3 in the outer medulla [11].

PP has been described in children who have suffered from significant family pathology with emotional disturbances or social neglect [12, 13]. Forced water ingestion has also been described as a form of child abuse [14–16]. Other more routine stressors such as jealousy from a newborn sibling have rarely been reported in the literature [17] and highlight the need for a broad differential when evaluating a toddler with polyuria and polydipsia. Our case describes a toddler who appears to come from a stable home and whose onset of symptoms developed after mom's return to work. One hypothesis regarding the etiology of PP in such children starts with an environmental stressor that occurs between a parent and a child; the child finds relief and/or comfort by continuously drinking [18], leading to polydipsia, and polyuria ensues. It may also stem from the development of an attachment to water as a transitional object if parents tend to offer a bottle at the first sign of distress [3].

The pathophysiology of primary or psychogenic polydipsia is not clearly understood, although several factors likely play a role, including endocrine disturbances, exacerbation of psychotic symptoms, and impulse control. In animal models, administration of dopamine agonists initiates drinking and dopamine receptor blockade inhibits drinking [19]. Increases in dopamine and/or dopamine receptors are hypothesized to cause psychotic symptoms in schizophrenic patients and provide a link between psychotic illness and polydipsia [20]. The association of polymorphisms in the dopamine D2 receptor gene with polydipsia in schizophrenic patients [21] also links the two disorders. Alternatively, increased AVP levels are associated with anxiety, depression, and other mood disorders [22], although the specific role of increased AVP levels in the pathophysiology of primary or psychogenic polydipsia is unclear.

Although acute water intoxication as a complication of PP is a concern, it is unlikely to occur in children due to efficient body-fluid regulation and nearly unlimited capacity to excrete water loads assuming renal function is normal. However, body-fluid regulation can be overwhelmed by an abrupt variation of daily water intake, or in some instances, treatment with DDAVP, thereby leading to acute water intoxication. When hyponatremia is present, renal or adrenal disease must be considered. Signs and symptoms of water intoxication include urinary and fecal incontinence, emesis, bizarre behavior, seizures, and respiratory arrest [15]. Water intoxication is more common in infants [14, 23] and is usually a result of improper feeding techniques with excessive free water administration or dilute formula.

## 4. Conclusion

Primary polydipsia is important to consider even in toddler-aged children presenting with polydipsia and polyuria. This diagnosis can be easily missed or misdiagnosed due to reluctance of practitioners to associate compulsive drinking behavior with otherwise nonpsychotic children. A formal water deprivation test is imperative in distinguishing it from diabetes insipidus. Differentiating PP from DI is important because treatment of PP with DDAVP can abolish the protective diuresis leading to potentially fatal fluid overload, profound hyponatremia, and impairment of water concentrating ability.

## Figures and Tables

**Table 1:** Causes of polyuria and polydipsia in children.

Primary polydipsia
Central diabetes insipidus
Nephrogenic diabetes insipidus
Obstructive uropathy
Renal failure
Conn's syndrome
Addisonian crisis
Diabetes mellitus
Hypokalemia
Hypercalciuria
Hypercalcemia
Bartter syndrome
Fanconi syndrome
Sickle cell anemia
Anorexia nervosa

**Table 2:** Interpretation of serum and urine osmolality in the differential diagnosis of polyuria and polydipsia after the water deprivation test.

Serum osmolality (mOsm/kg)	Urine osmolality after deprivation (mOsm/kg)	Urine osmolality after DDAVP (mOsm/kg)	Plasma AVP level	Diagnosis
>300	<300	>750	<2	Central DI
>300	<300	<300	>5	Nephrogenic DI
<290	>750	>750	2–5	Primary polydipsia
>290	300–750	<750	Variable	Partial DI or primary polydipsia

DDAVP, desmopressin; AVP, arginine vasopressin (pg/mL); DI, diabetes insipidus.

## Abbreviations

AVP: Arginine vasopressin
DDAVP: Desmopressin
CDI: Central diabetes insipidus
NDI: Nephrogenic diabetes insipidus
PP: Primary polydipsia.
